# Effects of Heterogenization Treatment on the Hot-Working Temperature and Mechanical Properties of Al-Cu-Mg-Mn-(Zr) Alloys

**DOI:** 10.3390/ma16124256

**Published:** 2023-06-08

**Authors:** Ming-Che Wen, Yuan-Da Hsu, Mien-Chung Chen, Wen-Chen Yang, Sheng-Long Lee

**Affiliations:** 1 Institute of Material Science and Engineering, National Central University, Taoyuan 320, Taiwan; j0e9rr3y0@gmail.com (M.-C.W.); mianzhongchen@gmail.com (M.-C.C.); wsntpe877@gmail.com (W.-C.Y.); 2 Department of Mechanical Engineering, National Central University, Taoyuan 320, Taiwan; vm3m06286@gmail.com

**Keywords:** Al-Cu-Mg-Mn alloy, zirconium, heterogenization, Al_3_Zr, melting temperature

## Abstract

This study investigated the effects of a minor Zr addition (0.15 wt%) and heterogenization treatment (one-stage/two-stage) on the hot-working temperature and mechanical properties in Al-4.9Cu-1.2Mg-0.9Mn alloy. The results indicated that the eutectic phases (α-Al + θ-Al_2_Cu + S-Al_2_CuMg) dissolved after heterogenization, retaining θ-Al_2_Cu and τ_1_-Al_29_Cu_4_Mn_6_ phases, while the onset melting temperature increased to approximately 17 °C. A change in the onset melting temperature and evolution of the microstructure is used to assess an improvement in hot-working behavior. With the minor Zr addition, the alloy exhibited enhanced mechanical properties due to grain growth inhibition. Zr-added alloys show 490 ± 3 MPa ultimate tensile strength and 77.5 ± 0.7 HRB hardness after T4 tempering, compared to 460 ± 2.2 MPa and 73.7 ± 0.4 HRB for un-added alloys. Additionally, combining minor Zr addition and two-stage heterogenization resulted in finer Al_3_Zr dispersoids. Two-stage heterogenized alloys had an average Al_3_Zr size of 15 ± 5 nm, while one-stage heterogenized alloys had an average size of 25 ± 8 nm. A partial decrease in the mechanical properties of the Zr-free alloy was observed after two-stage heterogenization. The one-stage heterogenized alloy had 75.4 ± 0.4 HRB hardness after being T4-tempered, whereas the two-stage heterogenized alloy had 73.7 ± 0.4 HRB hardness after being T4-tempered.

## 1. Introduction

Al-4.9Cu-1.2Mg-0.9Mn alloy (AA2024) is a heat-treatable wrought aluminum alloy known for its high strength and excellent fatigue resistance compared to other alloys in the 2xxx series [1]. However, hot-working of AA2024 aluminum alloy often presents challenges. The eutectic phases (α + θ + S) at the grain boundaries are difficult to eliminate through subsequent homogenization processes [2]. When the hot-working temperature exceeds the melting point of these eutectic phases, hot shortness can occur on the extruded surface [3]. The presence of remaining eutectic phases with low melting points further lowers the hot-working temperature range to 420–450 °C [4]. Additionally, grain coarsening can take place during high-temperature heat treatment, subsequently reducing the mechanical strength of Al-Cu-Mg alloys [5].

To increase the hot-working rate of AA2024 alloy, it was necessary to determine whether the eutectic phase could be dissolved during homogenization [2]. A conventional one-stage homogenization method dissolves eutectic phases in the aluminum matrix and redistributes the solute atoms, but it gives little attention to the effects of multiple procedures on the subsequent hot-working process [6]. The literature shows that the ternary eutectic phase (α + θ + S) can be effectively decreased by decreasing the Mg content, which can increase the melting temperature [7]. However, with a decrease in the Mg content, the amount of the strengthening phase (S′-Al_2_CuMg) can decrease, thereby decreasing the alloy’s strength [8]. In this study, the hot-working behavior takes reference from changes in the onset melting temperature and microstructure evolution.

Elements, such as Mn and Zr, are often added to aluminum alloys to inhibit recrystallization and grain growth during the solution treatment. Mn in Al-Cu-Mg alloys form long strip-shaped Al_20_Cu_2_Mn_3_ phases after the heat treatment [9,10,11]. Although the Al_20_Cu_2_Mn_3_ phase can improve the alloy strength, it cannot solve the problem of grain coarsening [12]. Previous works have evidenced that Al_3_Zr dispersoids formed in 7xxx series alloys via minor Zr addition can significantly improve the inhibition of recrystallization or grain growth, as well as the strength of the alloy [13,14,15,16,17]. Additionally, the two-stage heterogenization treatment produces finer and denser Al_3_Zr dispersoids which help to increase the recrystallization resistance in 7xxx series alloys [18,19,20].

The present study investigated the effects of one-stage and two-stage heterogenization on the hot-working temperature and mechanical properties of wrought Al-4.9Cu-1.2Mg-0.9Mn-(0.15Zr) alloy. Attention was paid to the alloy’s melting temperature after heterogenization and the mechanical properties after T4-tempered treatment. The heterogenization design enhanced the dissolution of eutectic phases and promoted the formation of fine Al_3_Zr dispersoids. The results can provide a reference for the development of high-strength 2xxx series aluminum alloys with higher hot workability and mechanical properties, whereas most related research focused solely on 7xxx series aluminum alloys.

## 2. Materials and Methods

### 2.1. Melting and Casting

High-purity aluminum ingots (99.9 wt.%) and master alloys were melted in a graphite crucible using an electric resistance furnace. Pure argon was used for degassing the melts for 30 min. The melts were then poured into a 300 °C preheated steel mold (125 mm × 100 mm × 25 mm). Two experimental alloys were used: Alloy A (0Zr) and Alloy B (0.15Zr). The chemical compositions of the experimental alloys, determined via inductively coupled plasma optical emission spectrometry (ICP-OES), are listed in Table 1.

### 2.2. Heat Treatment Cycle of the As-Cast Alloys

**Figure 1:**
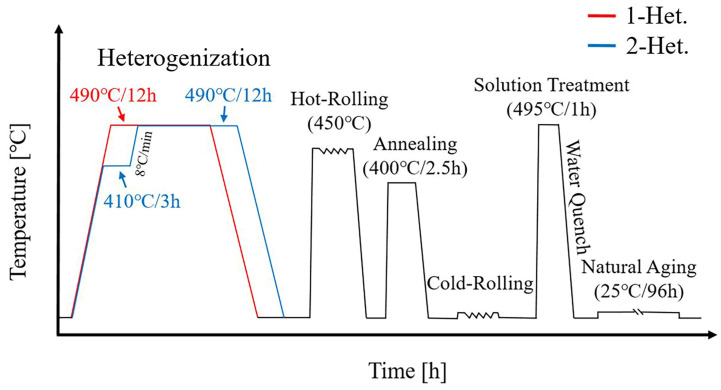
Heat treatment cycle schedule of the as-cast alloys.

Figure 1 shows the heat treatment cycle schedule for the as-cast alloys. Heterogenization of the casting alloys was divided into a one-stage treatment (1-Het.) and a two-stage treatment (2-Het.) One-stage heterogenization was performed at 490 °C for 12 h. Two-stage was firstly applied at 410 °C for 3 h, and the temperature was increased to 490 °C in 10 min then held for 12 h [21]. After heterogenization, the alloys were hot-rolled at 450 °C to 3 mm thick, annealed at 400 °C for 2.5 h in an air furnace, cold-rolled at room temperature to 2.25 mm thick, and then finally treated with T4 heat treatment. The T4 heat treatment was conducted at 495 °C for 1 h, followed by water quenching as solution treatment and then natural aging for 96 h [22].

### 2.3. Microstructure Characterization

Optical microstructure samples were ground by standard processes and then with electrolytic polishing using Barker’s reagent (2% HBF_4_) at 25 °C and with 20 V working voltage. Three-dimensional microstructures were constructed to recognize the grain structures of cold-rolled and solution-treated alloys. The rolling direction, transverse direction, and normal direction are denoted as RD, TD, and ND. EBSD specimens were electro-polished at 25 °C with a solution of 80 mL C_2_H_5_OH (99.5 wt.%) + 14 mL H_2_O + 6 mL HClO_4_ (70 wt.%), and the working voltage was 20 V [23]. EBSD data were analyzed using the software OIM Analysis™ V8, thus obtaining the grain size, aspect ratio, and misorientation angle distribution. To investigate the difference in recrystallization behavior, the low angle grain boundaries (LAGBs) were defined as the misorientation angles ranging from 2° to 15°, and those above 15° as were defined as high angle grain boundaries (HAGBs). The sum of the high angle grain boundaries’ fraction represents the recrystallization fraction. A SEIKO-DSC6200 (SEIKO, Chiba, Japan) differential scanning calorimeter at a heating rate of 10 °C/min was used to capture the DSC traces of the as-cast and heterogenized alloys. The electrical conductivities of the alloys were measured using a Sigma scope, SMP10 (FISCHER, Waldachtal, Germany). Microstructure observation and EBSD measurements were performed via JSM-7800F Prime scanning electron microscopy (JEOL, Tokyo, Japan) with a working voltage of 15 kV. The volume fraction of the α-Al and the second phase was determined by the software ImageJ V1.44. An electron probe microanalyzer (JEOL, JXA-8500F) was used to analyze the composition of the observed phases, and the applied working voltage was 12 kV. TEM specimens were prepared by cutting discs from the heterogenized samples, and they were then twin-jet electropolished in a mixture of 33% nitric acid and 67% methanol at −25 °C with a working voltage of 13 V. Afterwards, TEM observations were performed with JEOL JEM ARM200FTH.

### 2.4. Hardness and Tensile Testing

The hardness of the alloys was measured using the Rockwell hardness B scale (HRB) at a load of 100 kgf, and the application time for one indention was 5 s. Tensile tests were conducted with the MTS 810 Material Testing System, and with a constant crosshead speed of 0.2 mm/min. The yield stress was determined by the offset method at an offset of 0.2%. The specimens were prepared in accordance with the specimen specification in ASTM E8 standard [24].

## 3. Results and Discussion

### 3.1. Scanning Electron Microscope Observation of the As-Cast and Heterogenized Alloys

**Figure 2:**
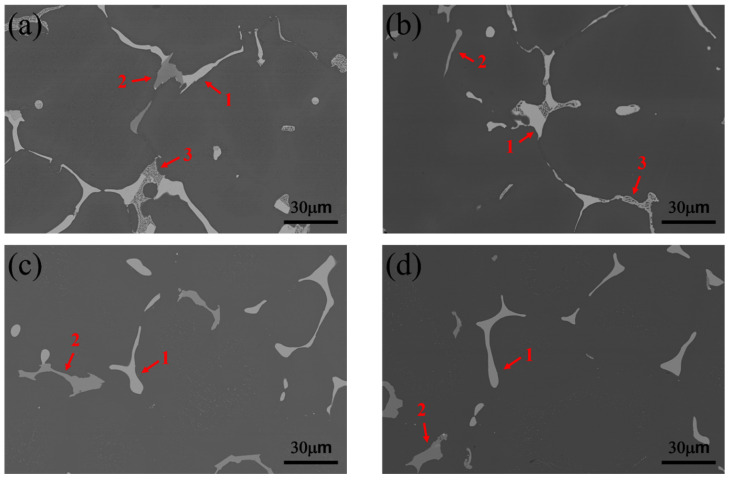
Microstructure observation by SEM-BEI and EPMA analysis of: (**a**) as-cast Alloy A; (**b**) as-cast Alloy B; (**c**) 1-Het. Alloy A; (**d**) 1-Het. Alloy B. (Arrow 1: eutectic θ-Al_2_Cu, 2: eutectic τ_1_-Al_29_Cu_4_Mn_6_, 3: ternary eutectic α + θ-Al_2_Cu + S-Al_2_CuMg).

SEM-BEI images of the as-cast Alloy A (0Zr) and Alloy B (0.15Zr) are shown in Figure 2a,b, respectively. Notably, there are three types of eutectic phases on the grain boundary of the as-cast alloys: the θ-Al_2_Cu phase, τ_1_-Al_29_Cu_4_Mn_6_ phase including impurities of Fe and Si, and ternary eutectic phase (α-Al + θ-Al_2_Cu + S-Al_2_CuMg) [25,26]. The eutectic phase mainly consisted of the θ-Al_2_Cu and ternary eutectic phases, whereas the amount of the τ_1_-Al_29_Cu_4_Mn_6_ phase was extremely low. The microstructures of Alloy A and Alloy B after the one-stage heterogenization treatment is shown in Figure 2c,d. It was found that most eutectic phases were dissolved, almost all ternary eutectic phases (α + θ + S) were dissolved in the Al matrix, and a few θ-Al_2_Cu and τ_1_-Al_29_Cu_4_Mn_6_ phases remained on the grain boundary. The volume fraction of the second phase (α-Al else) decreased from 5.9% to 2.9% in the Alloy A, while the volume fraction decreased from 6.2% to 3.1% in the Alloy B. However, although the two-stage heterogenization treatment (410 °C/3 h + 490 °C/12 h) includes a one-stage treatment (490 °C/12 h), the microstructure shows no significant difference. Two-stage heterogenization, however, shows no difference in SEM microstructure when compared with the one-stage heterogenized Alloy A and Alloy B. The microstructure consisted of α-Al, θ phases, ternary eutectic phases, and iron-rich phases. After heterogenization, the changes in volume fraction of the second phase were also around 3%. The literature reveals that the elimination of segregation and the dissolution of intermetallic particles have beneficial effects on the alloy’s further hot-working processing and development of mechanical properties [2]. These are related to the shape and type of intermetallic particles, a reduction in the flow stress of the material due to the precipitation of dispersoids or other precipitates, and the removal of micro-segregation [27]. 

### 3.2. Transmission Electron Microscope Observation of Heterogenized Alloys

**Figure 3:**
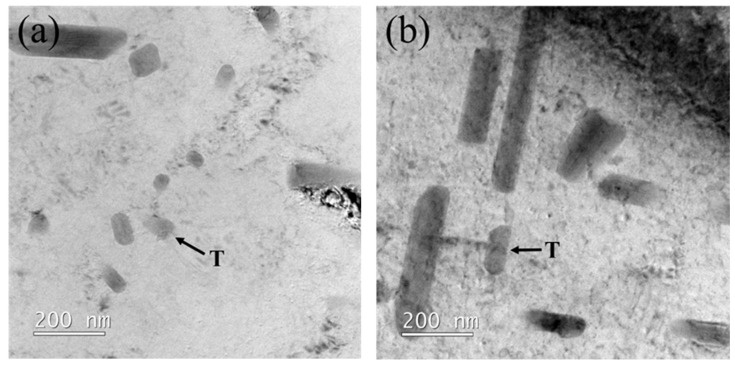

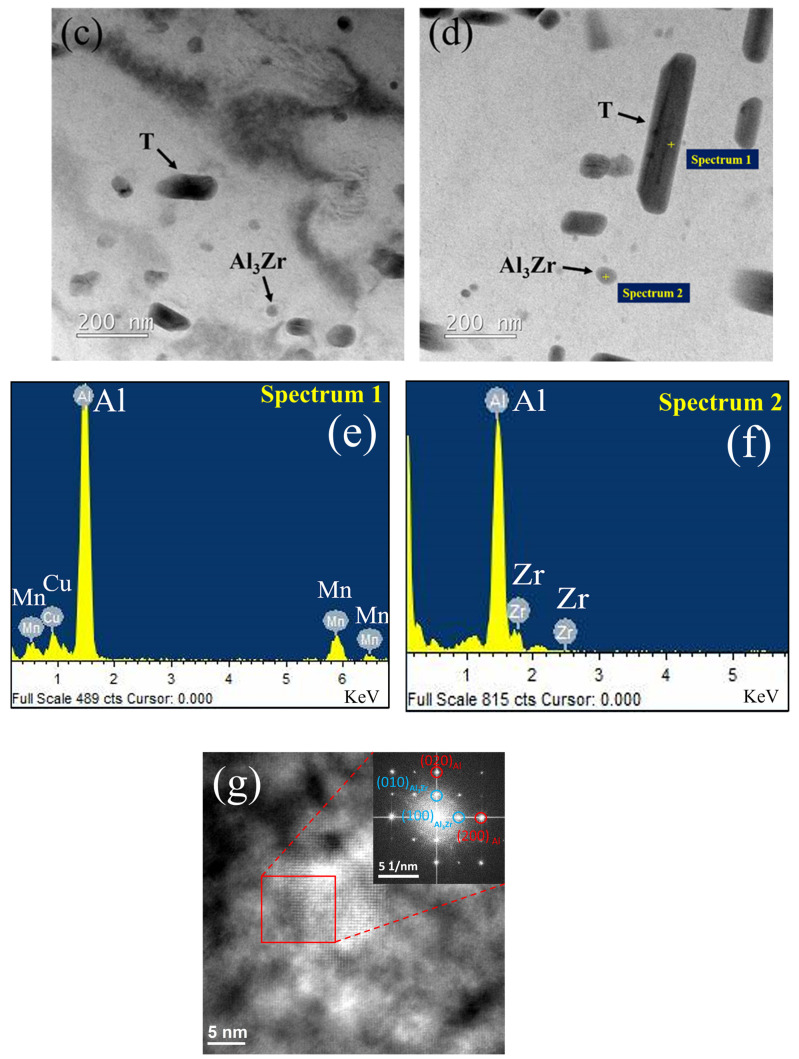
Microstructure observation showing T-phase (rod-like) and Al_3_Zr (spherical-like) by TEM analysis of: (**a**) 1-Het. Alloy A; (**b**) 2-Het. Alloy A; (**c**) 1-Het. Alloy B; (**d**) 2-Het. Alloy B, and EDS analysis results of: (**e**) T-phase, and (**f**) Al_3_Zr dispersoids, and also: (**g**) HR-TEM image along with the inset showing fast Fourier transform (FFT) pattern of the red line region.

TEM observations of the one-stage and two-stage heterogenized Alloy A and Alloy B are shown in Figure 3. The Al-4.9Cu-1.2Mg alloy with added Mn and Zr can precipitate T-Al_20_Cu_2_Mn_3_ phases and Al_3_Zr dispersoids on the Al matrix [10,11,12]. Alloy A, without Zr addition, precipitated the rod-shaped T-Al_20_Cu_2_Mn_3_ phases on the Al matrix, as shown in Figure 3a,b. In contrast, Alloy B, with Zr addition, precipitated fine Al_3_Zr dispersoids in addition to the rod-shaped T-Al_20_Cu_2_Mn_3_ phase, as shown in Figure 3c,d. Furthermore, it can be observed that the Al_3_Zr precipitates treated by the two-stage heterogenization are finer than those treated by the one-stage heterogenization. After the one-stage heterogenization, the average size of the Al_3_Zr particles was 25 nm, with a standard deviation of 8 nm. After the two-stage heterogenization, the average size of the Al_3_Zr particles was reduced to 15 nm, with a standard deviation of 5 nm. Similar results were observed by Guo [28] on 7150 alloy as, after two-step (300 °C/48 h + 470 °C/24 h) heat treatment the average radius of Al_3_Zr dispersoids was 10.7 nm. This difference in particle size may be attributed to the heat treatment temperature and duration [19,29]. The precipitation temperature of L1_2_ coherent Al_3_Zr ranges from 350 °C to 450 °C [21]. The dispersoids’ size mainly depends on two factors: (1) the supersaturation of solid solutions and (2) the diffusion rate of solute atoms. When heterogenized at a high temperature (490 °C), the supersaturation of Zr in solid solution is low, but Al_3_Zr dispersoids grow faster due to the high diffusion rate. On the other hand, heterogenization at a lower temperature leads to a greater driving force of nucleation due to increased Zr supersaturation. Additionally, the lower diffusion rate of Zr at a lower temperature helps to produce finer and denser Al_3_Zr dispersoids. The first stage of the two-stage heterogenization involves a precipitation temperature of L1_2_ coherent Al_3_Zr which acts as heterogeneous nucleation sites during higher temperature treatment, and thus helps to precipitate finer Al_3_Zr particles [21]. In contrast, one-stage heterogenization raises the temperature to 490 °C and maintains it for 12 h. Since the heat treatment temperature is significantly higher than the precipitation temperature of L1_2_ coherent Al_3_Zr and the duration is longer, the Al_3_Zr dispersoids grow larger [30]. Figure 3e,f presents the results of energy dispersive spectroscopy analysis of point spectrum 1 and point spectrum 2. The peaks of Cu and Mn confirm the presence of rod-like phases as T-Al_20_Cu_2_Mn_3_, while the peaks of Zr indicate the presence of spherical-like particles as Al_3_Zr dispersoids. Figure 3g presents the high-resolution transmission electron microscopy (HR-TEM) image along with the fast Fourier transform (FFT) pattern, taken from the <001> zone axis, which revealed that the aluminum matrix and Al_3_Zr dispersoids had an ordered of L1_2_ structure [31].

### 3.3. Optical Microstructure of Cold-Rolled and T4-Tempered Alloys

**Figure 4:**
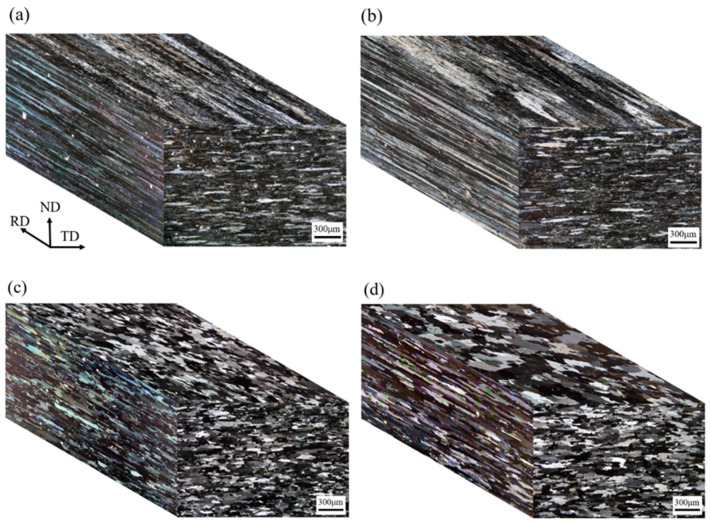
Three-dimensional optical microstructure of the Zr-free Alloy A: (**a**) 1-Het. and then cold-rolled; (**b**) 2-Het. and then cold-rolled; (**c**) 1-Het. and then T4-treated; (**d**) 2-Het. and then T4-treated.

**Figure 5:**
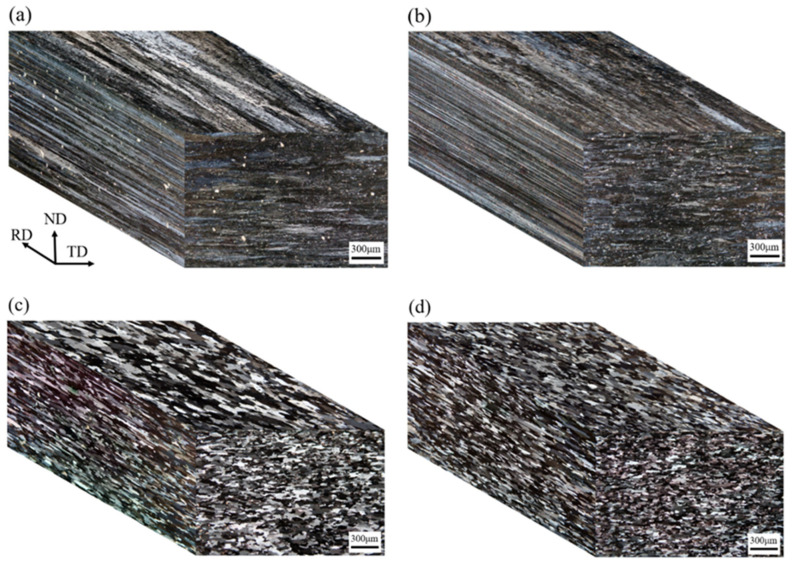
Three-dimensional optical microstructure of the Zr-added Alloy B: (**a**) 1-Het. and then cold-rolled; (**b**) 2-Het. and then cold-rolled; (**c**) 1-Het. and then T4-treated; (**d**) 2-Het. and then T4-treated.

The microstructure of Alloy A (0Zr) after one-stage/two-stage heterogenization and cold-rolling is shown in Figure 4a,b. The rolling, transverse, and normal directions are denoted as RD, TD, and ND, respectively. The microstructure in the cold-rolled state showed no significant difference when applying heterogenization from one-stage to two-stage. The microstructure of the T4-tempered Alloy A is shown in Figure 4c,d, revealing a high recrystallization fraction in the grains. The average grain size, aspect ratio, and recrystallization fraction are listed in Table 2. It can be observed that for Alloy A, whether heterogenized by one-stage or two-stage treatment, the grains exhibit a significant preferred orientation with a tendency to grow in the rolling direction. The aspect ratios are 4.7 and 5.8, respectively. This phenomenon is attributed to the presence of fine T-Al_20_Cu_2_Mn_3_ dispersed particles along the rolling direction, which inhibit grain growth in the vertical direction [17]. Furthermore, the grain growth in the two-stage heterogenized alloy is more pronounced than in the one-stage heterogenized alloy, indicating that the dispersed phases of T-Al_20_Cu_2_Mn_3_ formed by two-stage heterogenization have a weaker ability to inhibit grain growth compared to alloys with one-stage heterogenization. The microstructures of Alloy B (0.15Zr), heterogenized by one-stage/two-stage and then cold-rolled, are shown in Figure 5a,b. The grain size in the two-stage heterogenization is finer than in the one-stage heterogenization, suggesting that the fine Al_3_Zr dispersoids are more effective than the T-Al_20_Cu_2_Mn_3_ phases in resisting the movement of grain boundaries. The microstructures of the T4-tempered Alloy B are shown in Figure 5c,d, with either one-stage or two-stage heterogenization, and the grains present a high recrystallization structure.

### 3.4. EBSD Analysis of the T4-Tempered Alloys

**Figure 6:**
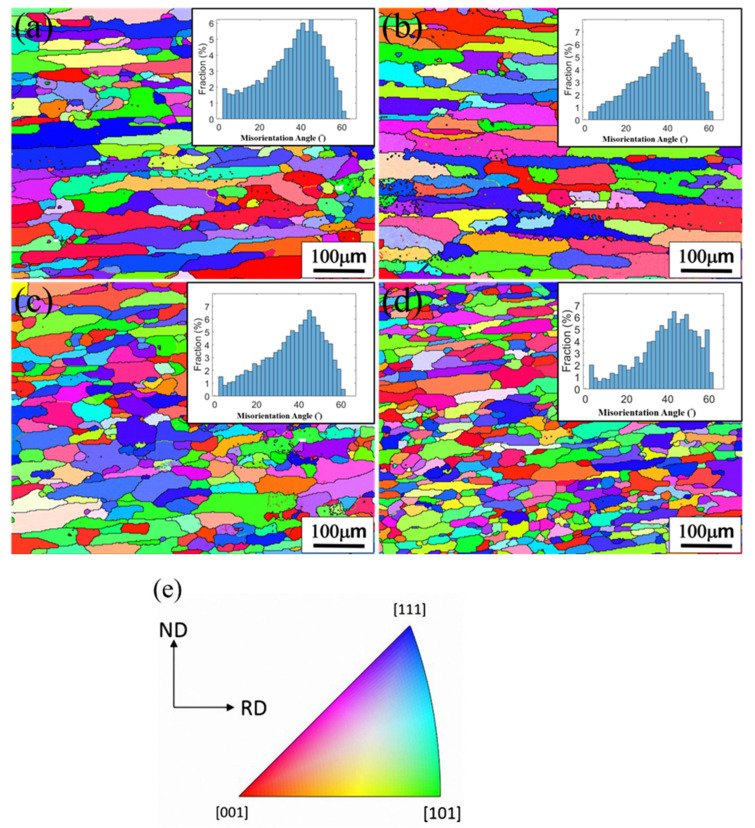
Euler angle colored EBSD maps and misorientation angle distribution of T4 state of: (**a**) 1-Het. Alloy A; (**b**) 2-Het. Alloy A; (**c**) 1-Het. Alloy B; (**d**) 2-Het. Alloy B; (**e**) IPF figure.

Figure 6 shows the EBSD analysis results of the two T4-state alloys, heterogenized by one-stage (1-Het.) and two-stage treatment (2-Het.). The data are summarized in Table 2. The results demonstrate no significant difference in the recrystallization fraction of the T4-state alloy, which is related to Zr addition or two-stage heterogenization. The high recrystallization fraction (90%) is attributed to the fact that the recrystallization temperature (400–450 °C) [32], which is enhanced by Al_3_Zr particles, is lower than the solution-treatment temperature of AA2024 (495 °C). As a result, all cold-rolled alloys were almost fully recrystallized after the high-temperature solution treatment. Similarly, Alloy B treated by two-stage heterogenization can effectively inhibit grain growth after recrystallization, whereas Alloy A shows the opposite. This result demonstrates that the fine Al_3_Zr dispersed phase has a better capability to inhibit the movement of the grain boundaries and produces the finest grains in the T4-state alloy.

### 3.5. DSC Analysis of the As-Cast and Heterogenized Alloys

**Figure 7:**
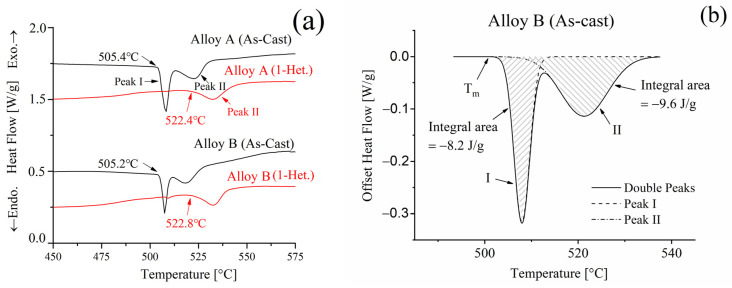
DSC traces of the as-cast and one-stage heterogenization Alloy A(0Zr) and Alloy B(0.15Zr). (**a**) DSC curve; (**b**) Gaussian fitting of the as-cast Alloy B.

An enlarged view of the DSC traces of the as-cast and heterogenized alloys from 450 to 575 °C is presented in Figure 7a. The first endothermic peak (Peak I) between 505 and 515 °C corresponds to the melting of the ternary eutectic phases (α + θ + S). The second endothermic peak (Peak II) between 510 and 530 °C corresponds to the melting of the binary eutectic phases (α + θ) [5,28,29,30,31,32,33]. Owing to the absence of ternary eutectic phases (α + θ + S), the melting temperature increases by 17 °C in the one-stage heterogenized condition compared to the as-cast condition. Using analysis software (Origin Pro V9.0) and the Gaussian fitting method, the enthalpy change (Q) of Peak I and Peak II is calculated as −8.2 (J/g) and −9.6 (J/g), just as shown in Figure 7b. The absolute enthalpy change (|Q|) was used as a reference for the eutectic phase [16]. The absolute enthalpy changes of the as-cast (|Q*_ac_*|), one-stage heterogenized (|Q*_h_*|) and the elimination percentage of the eutectic phase [(|Q*_h_*| − |Q*_ac_*|)/|Q*_ac_*| × 100%] are summarized in Table 3. After the one-stage heterogenization (490 °C/12 h), the absolute enthalpy changes (|Q*_h_*|) of Alloy A (0Zr) and Alloy B (0.15Zr) were only 0.05 J/g. The results indicate that the ternary eutectic phase (α + θ + S) of Alloy A and Alloy B is mostly eliminated after the one-stage heterogenization treatment. Additionally, it can be observed that the elimination percentage was greater than 99%. Therefore, the onset melting temperature (T_m_) of Alloy A and Alloy B increased to 522.4 and 522.8 °C, respectively, which is approximately 17 °C higher than that of the as-cast alloys. The DSC curve of the alloy after two-stage heterogenization is similar to that after one-stage heterogenization and is consistent with the microstructural observations shown in Figure 2.

### 3.6. Hardness and Tensile Properties

**Figure 8:**
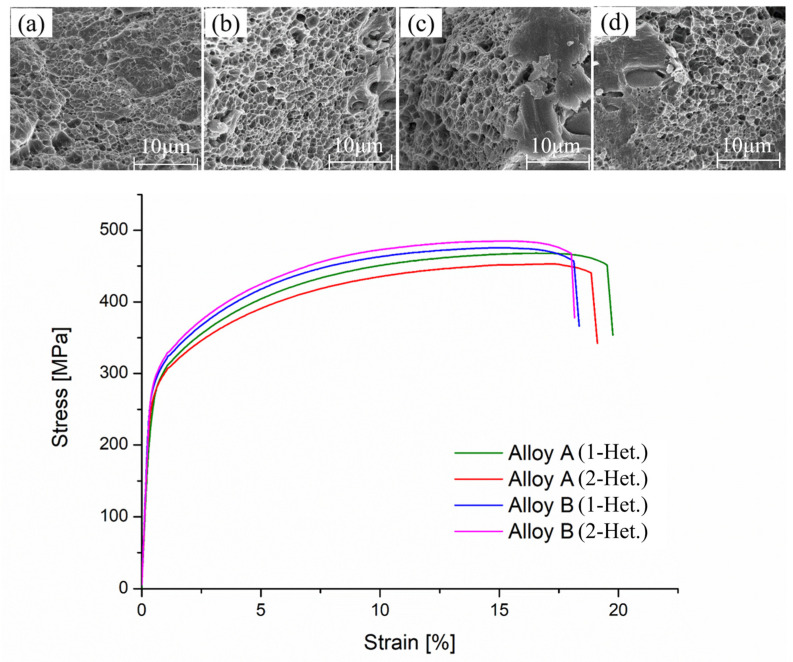
Representative engineering stress-strain curve and fracture micrographs of the experimental alloys after T4 heat treatment. (**a**) Alloy A (1-Het.); (**b**) Alloy A (2-Het.); (**c**) Alloy B (1-Het.); (**d**) Alloy B (2-Het.).

Typical engineering stress-strain curves and fracture micrographs of the experimental alloys after the T4 heat treatment are shown in Figure 8. The yielding stress (YS), ultimate tensile stress (UTS), elongation (EL), and hardness are summarized in Table 4. The precipitation behavior of Al-Cu-Mg alloys at room temperature has been well reported. Ivanov [34] used small-angle neutrons and X-ray scattering (SANS and SAXS) to extract the chemically resolved kinetics of room temperature clustering in the Al-1.1Cu-1.7Mg alloy, and the results clearly identify Mg as a solute required for clustering in the Al-Cu system at room temperature, where aging for 72 h after quench is considered stable and which has 117 ± 3 Hv (~62.3 HRB) hardness. In our study, after one-stage heterogenization, the hardness of Alloy A aged for 96 h was 75.4 ± 0.4 HRB, while for two-stage heterogenization, the hardness of Alloy A aged for 96 h was 73.7 ± 0.4 HRB. Deng [35] investigated the effects of Al_3_Sc_1−x_Zr_x_ nanoparticles on the mechanical properties of aged Al-5.2Zn-1.82Mg alloy and found that the Al_3_Sc_1−x_Zr_x_ particles improved the yield strength by 161 ± 7 MPa (40 ± 3 percent) and increased the ultimate tensile strength by 122 ± 4 MPa (27 ± 1 percent). Due to the addition of Zr, Alloy B exhibited a relatively higher hardness of around 76.5 ± 0.4 HRB. With two-stage heterogenization, the hardness further improved to 77.5 ± 0.7 HRB. Similarly, alloys with Zr addition and which were treated with two-stage heterogenization show better tensile properties. The YS of Alloy B increased from 286 ± 4.6 MPa to 295 ± 2.0 MPa, evidencing that the precipitation of dense Al_3_Zr can enhance the mechanical properties of alloys during two-stage heterogenization. This result is consistent with the DSC results. Owing to the dense Al_3_Zr precipitates and finest grain size, the two-stage heterogenized Alloy B exhibits the greatest hardness and tensile strength among all the experimental alloys after T4 heat treatment. Notably, the hardness and tensile strength of the Alloy A treated with the two-stage heterogenization were lower compared to those of the one-stage heterogenized alloy. The mechanical properties of Zr-free Al-4.9Cu-1.2Mg-0.9Mn alloys cannot be improved by two-stage heterogenization. The decrease in mechanical properties may be caused by coarsening of the T-Al_20_Cu_2_Mn_3_ phase. The literature reveals that the formation temperature of the T-Al_20_Cu_2_Mn_3_ phase is between about 300 °C and 350 °C [36]. With applied two-stage heterogenization (410 °C/3 h + 490 °C/12 h), the T-Al_20_Cu_2_Mn_3_ phase which forms in the early stage (410 °C/3 h) would coarsen in the following stage (490 °C/12 h). These results are consistent with Figure 3b–d, where two-stage heterogenization resulted in a coarser T-Al_20_Cu_2_Mn_3_ phase. The fracture micrographs of the alloys after the T4 heat treatment, as shown in Figure 8a–d, reveal typical ductile fracture surfaces with shallow dimples. Additionally, a small area of brittle transgranular fracture surface and cleavages can be observed in the alloys with Zr addition.

## 4. Conclusions

The effects of minor Zr addition (0.15 wt%) and heterogenization (one-stage/two-stage) on the hot-working temperature and mechanical properties of Al-4.9Cu-1.2Mg-0.9Mn alloy were investigated and the following conclusions can be drawn:

(1) Through heterogenizing heat treatment, the eutectic phases (α + θ + S) could dissolve into the α-matrix, with an elimination percentage greater than 99%. As a result, the onset melting temperature of the heterogenized alloy decreased by 17 °C compared to the as-cast alloy, thereby increasing the hot-working temperature of the Al-4.9Cu-1.2Mg-0.9Mn alloy.
(2) Compared with one-stage heterogenization, two-stage heterogenization produces finer and denser Al_3_Zr dispersoids. The average size of Al_3_Zr decreased from 25 ± 8 nm to 15 ± 5 nm, effectively inhibiting grain growth during T4 tempering and resulting in improved mechanical properties.
(3) The Al-4.9Cu-1.2Mg-0.9Mn alloy, with a minor Zr addition and treated by two-stage heterogenization, exhibited the best hot workability and the highest mechanical properties. The melting temperature increased to 522.8 °C. Additionally, the hardness, yield strength, and ultimate tensile strength of the T4-aged alloy subjected to two-stage heterogenization increased to 77.5 ± 0.7 HRB, 295 ± 2.0 MPa, and 490 ± 3.0 MPa, respectively.

## Figures and Tables

**Table 1:** Chemical composition of experimental alloys (wt.%).

Alloy	Cu	Mg	Mn	Zr	Fe	Si	Al
A (0Zr)	4.91	1.22	0.92	-	0.09	0.04	Balance
B (0.15Zr)	4.88	1.21	0.91	0.16	0.09	0.04	Balance

**Table 2:** Average grain size (μm), aspect ratio and recrystallization fraction (%) of the one/two stage heterogenized Alloy A and Alloy B after T4 treatment.

Notation.	RD	ND	Aspect Ratio	Recrystallization Fraction
Alloy A (1-Het.)	117.8 (45.5) *	24.9 (3.4)	4.7 (0.6)	90.84 (0.8)
Alloy A (2-Het.)	163.8 (50.2)	28.3 (3.5)	5.8 (0.4)	90.40 (1.0)
Alloy B (1-Het.)	67.3 (15.8)	23.7 (2.8)	2.8 (1.0)	90.15 (1.2)
Alloy B (2-Het.)	57.0 (19.8)	22.3 (3.2)	2.5 (0.7)	91.16 (2.0)

* Standard deviations are listed in parentheses.

**Table 3:** Absolute enthalpy changes (|Q|, J/g) and elimination percentage (%) of the eutectic phases of the as-cast and one-stage heterogenized alloys.

Notation	Peak I (α + θ + S)			Peak II (α + θ)		
	|Q*_ac_* |	|Q*_h_*|	(*|Q_h_|* − *|Q_ac_|*)/ *|Q_ac_|* × 100%	|Q*_ac_*|	|Q*_h_*|	(*|Q_h_|* − *|Q_ac_|*)/ *|Q_ac_|* × 100%
Alloy A	7.9 (0.1) *	0.05 (0.003)	–99.3% (0.2)	8.5 (0.1)	5.3 (0.1)	–37.6% (0.5)
Alloy B	8.2 (0.1)	0.05 (0.005)	–99.4% (0.3)	9.6 (0.2)	5.9 (0.1)	–38.5% (1.0)

* Standard deviations are listed in parentheses.

**Table 4:** Hardness and tensile properties of the experimental alloys after T4 heat treatment.

Notation	Hardness (HRB)	YS (MPa)	UTS (MPa)	EL (%)
Alloy A (1-Het.)	75.4 (0.4) *	284 (2.0)	466 (2.8)	19.4 (0.5)
Alloy A (2-Het.)	73.7 (0.4)	280 (3.5)	460 (2.2)	19.5 (0.4)
Alloy B (1-Het.)	76.5 (0.4)	286 (4.6)	477 (5.5)	18.8 (0.4)
Alloy B (2-Het.)	77.5 (0.7)	295 (2.0)	490 (3.0)	18.0 (0.2)

* Standard deviations are listed in parentheses.

## Data Availability

Data available on request due to privacy restrictions.
